# Maximizing the Carrier Mobilities of Metal–Organic Frameworks Comprising Stacked Pentacene Units

**DOI:** 10.1021/acs.jpclett.1c01892

**Published:** 2021-07-20

**Authors:** Egbert Zojer, Christian Winkler

**Affiliations:** Institute of Solid State Physics, Nawi Graz, Graz University of Technology, Petersgasse 16, 8010 Graz, Austria

## Abstract

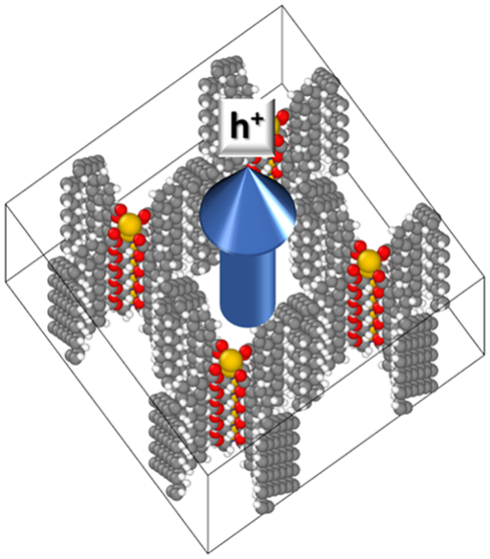

Charge transport properties of metal–organic frameworks (MOFs) are of distinct interest for (opto)electronic applications. In contrast to the situation in molecular crystals, MOFs allow an extrinsic control of the relative arrangement of π-conjugated entities through the framework architecture. This suggests that MOFs should enable materials with particularly high through-space charge carrier mobilities. Such materials, however, do not yet exist, despite the synthesis of MOFs with, for example, seemingly ideally packed stacks of pentacene-bearing linkers. Their rather low mobilities have been attributed to dynamic disorder effects. Using dispersion-corrected density functional theory calculations, we show that this is only part of the problem and that targeted network design involving comparably easy-to-implement structural modifications have the potential to massively boost charge transport. For the pentacene stacks, this is related to the a priori counterintuitive observation that the electronic coupling between neighboring units can be strongly increased by increasing the stacking distance.

Metal–organic frameworks (MOFs) are highly porous materials consisting of inorganic nodes connected by organic linkers.^1−3^ They are traditionally employed in fields like gas storage,^4−6^ catalysis,^7−9^ and gas separation.^10,11^ More recently, the electronic properties of MOFs have shifted into the focus of interest^12−14^ for applications including electrocatalysis,^15−19^ chemiresistive sensing,^20−25^ and energy storage.^26−28^ Of particular interest in this context are MOFs containing conjugated building blocks that are also used in the field of organic semiconductors. Here, MOFs hold the promise of enabling an improved π-stacking compared to the spontaneous self-assembly of conjugated molecules and, thus, improved *through-space* transport properties.^12,13,29^ Interesting examples for such systems comprise MOFs formed from linkers containing tetrathiafulvalene (TTF)^30−32,12,33^ or pentacene^34^ units. The crucial advantage is that in such MOFs the relative arrangement of the π-systems can be controlled via the network structure rather than following from the direct interactions of the conjugated units. This is insofar relevant, as interactions between π-conjugated materials favor packings with unfavorable transport properties, as this also minimizes intermolecular exchange repulsion.^35−37^ Suitably designed MOFs have the potential to overcome this driving force. Nevertheless, the through-space charge carrier mobilities of MOFs are typically ≪1 cm^2^V^–1^s^–1^. In a recent work on pentacene-containing MOFs, this has been attributed to dynamic disorder,^38−40^ as a consequence of frustrated rotations of the pentacene units.^34^ While such effects certainly play a decisive role for charge transport in the intermediate coupling regime often encountered in π-conjugated materials,^38,41,42^ we will argue here that for the MOFs studied in ref (34) another complication is that the framework structure enforces a packing of the pentacene units that is far from ideal for the finally obtained electronic coupling. Based on the insights from this analysis, we then propose a comparably straightforward to implement design strategy, which (relying on existing chemical building blocks) has the potential to increase the achievable carrier mobilities by roughly an order of magnitude (as demonstrated for the limiting cases of band- and hopping transport).

To analyze the electronic contribution to charge transport in MOFs comprising stacks of pentacene-units, we calculated various transport-relevant parameters (like bandwidths, effective masses, transfer integrals, and relative hopping rates) using density-functional theory (DFT) in conjunction with periodic boundary conditions. We employed the FHI-AIMS code,^43−46^ and for most calculations, we chose the Perdew–Burke–Ernzerhof (PBE) functional^47,48^ combined with a nonlocal variant of the many-body van der Waals correction.^49^ As shown in the Supporting Information, calculations on selected systems with the hybrid Heyd–Scuseria–Ernzerhof (HSE06)^50^ functional yielded equivalent results. To be able to consider the crystalline environment of the molecules, the transfer integrals were directly extracted from the band structures employing the tight-binding based approach described in ref (51) (with further details on the employed methodology provided in the Supporting Information).

The base structure of the pentacene MOF in the current study is the one suggested in ref (34). As shown schematically in Figure 1a, it is a variant of MOF-2 in which Zn-oxo paddlewheel nodes (blue spheres) are connected by four 6–13-substituted pentacene linkers (see Figure 1b). Atomistic representations of the optimized structures are contained in panels (c)–(e). These MOFs consist of pores, which are each surrounded by four stacks of pentacene units (red rectangles in Figure 1a). The structure with intact Zn-paddlewheels and four identical stacks around each pore (which has also been suggested in ref (34)) in the following will be referred to as the “paddle” structure. In this structure, all Zn atoms are aligned along straight lines parallel to **a**_**3**_ (see Figure 1d). Of particular relevance for the following discussion is the lattice constant a_3_, which also determines the distance between the centers of neighboring pentacene units (the stacking distance) and, concomitantly, the relative alignment of the pentacenes expressed by the π-slip and π-distance (see Figure 1a,d). For the paddle structure, a_3_ amounts to 5.78 Å, which is in excellent agreement with the experimentally determined lattice constant a_3_ of 5.8 Å.^34^ In a gedankenexperiment, this lattice constant will be varied while simultaneously optimizing the atomic positions, to analyze the impact of the stacking distance on the transport-relevant parameters. In passing, we note that in the course of these optimizations, also two other polymorphs of the pentacene-MOF were found and their structures were fully optimized (including unit-cell lengths; see Supporting Information). In one of them, the linkers running in **a**_1_ direction and those running in **a**_2_ direction are shifted by ca. one Zn–Zn distance parallel to **a**_**3**_, such that all Zn atoms in a column are connected by the carboxylate groups of two linkers for each pair (see Figure 1e). The a_3_ lattice constant of this “zipper” structure amounts to 6.09 Å. This is somewhat larger than that of the “paddle” conformation and the experimental value. The “zipper” structure is incompatible with the above-described gedankenexperiment, but as it represents the lowest-energy conformation, data for this polymorph will also be included in the following discussion. It is worth stressing that the properties calculated for the “zipper” polymorph and a third strongly distorted polymorph (see Supporting Information) align along the a_3_-dependent evolution obtained for the “paddle” derivatives; that is, the impact of these polymorphs on transport-relevant parameters can be directly traced back to differences in the respective stacking distances, a_3_.

**Figure 1:**
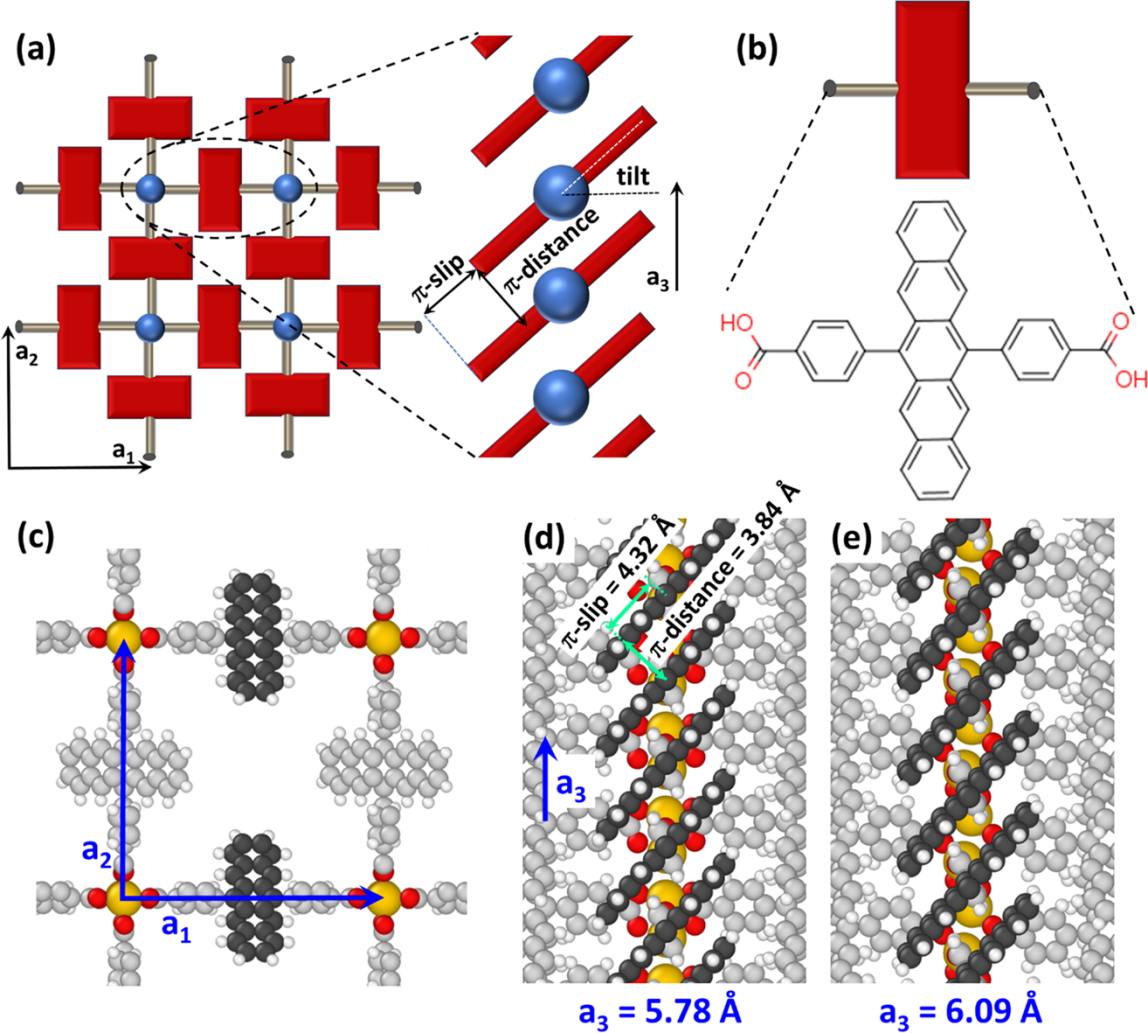
Structures of the studied pentacene-MOF. Panel (a) shows a schematic structure of the studied MOF with the blue spheres representing the Zn-paddlewheels and the actual structure of the linker molecules shown in panel (b). The right plot in panel (a) illustrates the structure of an individual stack of pentacene units with the stacking distance determined by the lattice constant a_3_. Panel (c) shows the atomistic representation of the optimized “paddle” geometry, illustrating the quadratic pores surrounded by four stacks of pentacene units. Panels (d) and (e) illustrate the atomistic structure of the pentacene stacks for the “paddle” and “zipper” polymorphs. Color code: dark and light gray: C; white: H; red: O; green: N; yellow: Zn.

As pentacene-based systems are typically employed as hole conductors due to their rather small ionization energies, the structures of the valence bands (VBs) are typically more relevant, but for illustrative purposes, in the following, we will also report conduction-band properties. As the unit cells of all studied systems are orthorhombic, the same applies also to the respective first Brillouin zones. In ΓZ direction (i.e., parallel to **a**_**3**_ in real space), the frontier bands in all systems with nonvanishing bandwidths display a cosine shape. This eases the extraction of transfer integrals employing the above-mentioned tight-binding model. All valence bands calculated for different stacking distances are shown in Figure 2a, where the thick black line corresponds to the situation of the equilibrium “paddle” geometry. All other relevant bands are shown in the Supporting Information, which also provides a detailed discussion including cases in which next-nearest neighbor couplings have a non-negligible impact on band shapes. Bands in ΓY and ΓX directions are flat (bandwidths ≤2 meV for the “paddle” polymorph) as a consequence of a very small electronic coupling in **a**_**1**_ and **a**_**2**_ directions due to confinement of the relevant orbitals to the pentacene units.

**Figure 2:**
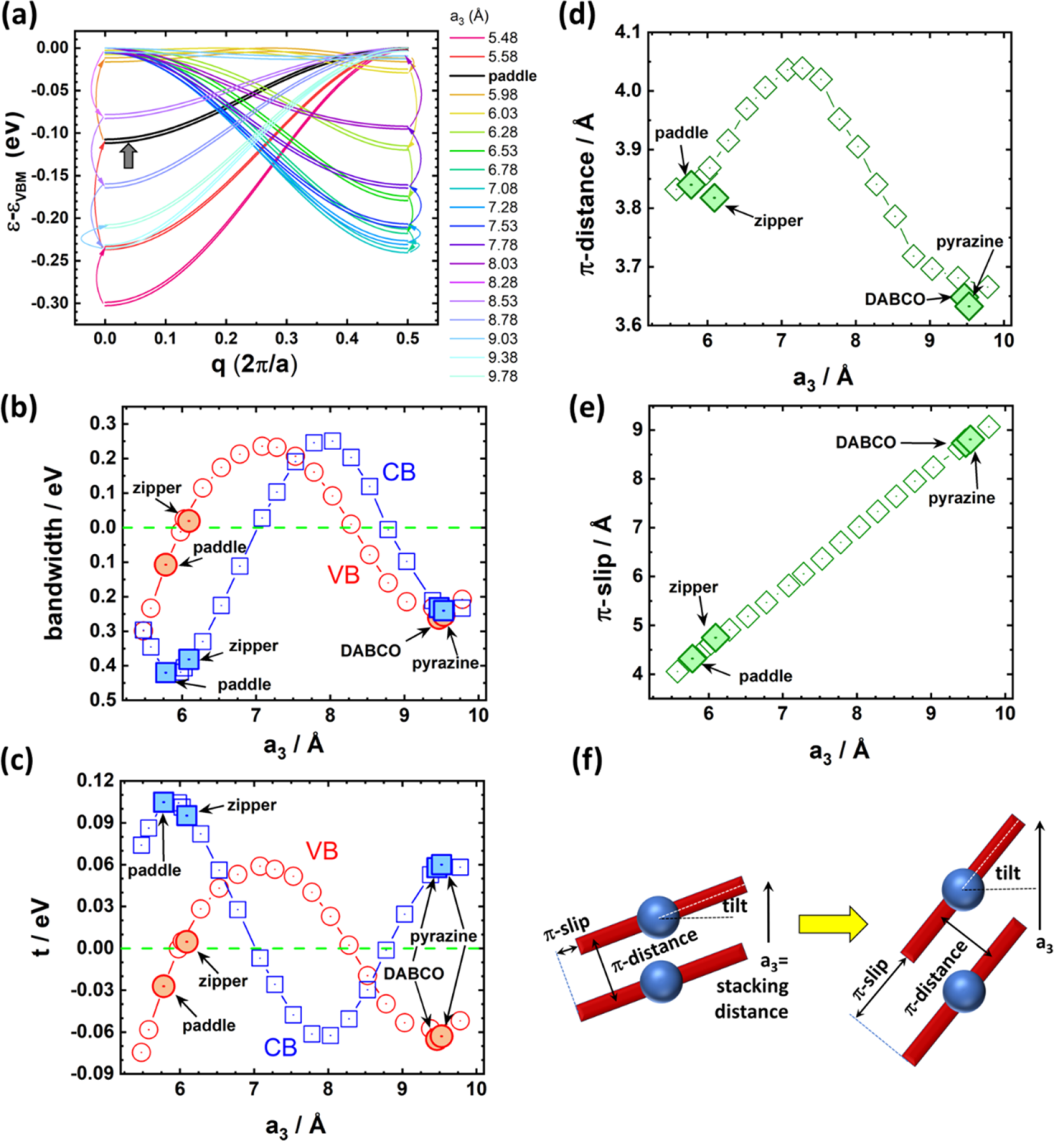
(a) Dependence of the structure of the valence band on the stacking distance expressed by the length of the lattice vector a_3_. As there are two pentacene units per unit cell, two close-lying and nearly degenerate bands are formed, as discussed in more detail in the Supporting Information. The bands of the “paddle” polymorph are shown by the thick black lines and are highlighted by the arrow. Panels (b) and (c) show the evolutions of the widths and associated transfer integrals, t, for the valence (red) and conduction bands (blue) along ΓZ direction (i.e., the direction in reciprocal space parallel to the pentacene stacking direction defined by **a**_**3**_) upon varying the stacking distance a_3_ in the model system described in the main text. The values for the “paddle”, “zipper”, “pyrazine”, and “DABCO” structures (the latter two discussed later in the manuscript) are also shown as larger, shaded symbols. One can see that they align along the evolution of the bandwidths and transfer integrals of the model system. Bandwidths are plotted above the zero line, in cases in which the valence band maxima (or conduction band minima are at the Γ-point). Otherwise, they are plotted below the zero line. In this way, also the positions of the band extrema are encoded into the plot. Conversely, the signs of the transfer integrals are a direct consequence of the Fourier cosine series ansatz of the tight-binding model (see Supporting Information). Panel (d) and (e) show the evolution of the π-stacking distance between neighboring pentacene planes and the π-slip (see Figure 1) as a function of the stacking distance given by a_3_. Panel (f) illustrates how the packing of neighboring pentacene units changes when the stacking distance is increased: While the π-distance is hardly modified, one observes a significant increase of the π-slip.

Interestingly, the stacking distance, a_3_, (varied in the above-described gedankenexperiment) has a huge impact on the calculated band shapes (Figure 2a) in terms of the positions of the band extrema and the bandwidths. The latter are shown with the associated transfer integrals in Figure 2b,c (open symbols). For the shortest considered stacking distance (5.48 Å), we observe the largest band dispersion and transfer integral for the valence band with a band maximum at the Z-point. Upon increasing a_3_, the absolute bandwidth, W, first decreases until it vanishes at a_3_ ≈ 6 Å. Beyond that the VB maximum lies at the Γ-point (see Figure 2a), first increases with a_3_ and then decreases again, until the band once more becomes flat around 8.3 Å. For larger values of a_3_, the band maximum switches back to the Z-point and the bandwidth again increases. The same evolution is obtained for the transfer integral, t, in Figure 2b, with W ≈ 4·t. The data points for the fully optimized “paddle” and “zipper” polymorphs are perfectly aligned with the discussed evolution. The width of the conduction band and the associated transfer integral also displays a pronounced dependence on a_3_, albeit with shifted zero-crossings and maxima.

To understand how an increase in the stacking distance, a_3_, can result in an increase of the transfer integral, one first has to analyze the variation of the geometry of the model MOFs. Importantly, the (normal) distance between the planes of neighboring pentacene units (denoted as π-distance in Figure 1) varies only rather weakly with a_3_. As shown in Figure 2d, it amounts to 3.84 Å for the parent “paddle” structure, increases to 4.04 Å for a_3_ = 7.1 Å (i.e., in the region of the pronounced maximum of the valence-bandwidth), and then drops to 3.67 Å for a_3_ = 9.8 Å. The reason for this evolution is that the equilibrium distance results from an interplay between van der Waals attraction (which drops with diminishing lateral overlap of neighboring pentacenes), Pauli repulsion (which also depends on the overlap and essentially scales with the widths of the occupied bands),^36^ and Coulombic interaction (mostly attractive due to charge penetration effects),^53−55^ which again drop with the overlap of neighboring molecules.^36,37^ These factors then typically play out such that the intermolecular overlap has only rather little impact on the equilibrium distance and, consequently, the equilibrium distance changes only slightly upon increasing the stacking distance (see schematic drawing in Figure 2e). This triggers a continuous increase of the tilt angle of the π-planes relative to the **a**_**1**_,**a**_**2**_-plane from 55.2° to 68.0° for the considered range of a_3_ values, as shown in the Supporting Information. As a consequence, the π-slip between the centers of consecutive pentacene units in the direction parallel to the π-planes increases essentially linearly from 4.06 to 9.07 Å over the considered range of a_3_ values (see Figure 2e); that is, the observed change in the π-slip is more than an order of magnitude larger than that of the π-distance.

An increase in the π-distance results in a decrease of the transfer integral due to the decreased wave function overlap, as illustrated for a cofacial pentacene dimer in the Supporting Information. The change in π-slip has, however, a much stronger impact on the band with and the transfer integral, as originally shown by Hofmann et al.^56^ and later discussed for a variety of organic semiconductors.^36,57−59,41,60^ This can be directly inferred from a comparison of Figure 2b,c with Figure 2d: it reveals that the maximum for the transfer integral and the bandwidth of the valence band occurs around a_3_ = 7 Å, where the distance between the pentacene planes actually reaches its maximum.

To illustrate the origin of the pronounced dependence of bandwidths and transfer integrals on the π-slip, Figure 3 shows the electronic states at the Γ and Z points of the “paddle” structure for the valence band (panel a) and for the conduction band (panel b). The situation is most obvious for the conduction band: For the electronic state at Z the phase of the wave function is switched between neighboring pentacenes. Consequently, at a stacking distance of 5.78 Å (the equilibrium value of the “paddle” phase), this state is described by a fully bonding linear combination of the molecular LUMOs of neighboring pentacene units (see white arrows), and it experiences a maximum energetic stabilization. Conversely, the Γ-point state (with the same phase of the wave function in each unit cell) represents a fully antibonding linear combination (see green arrows) and is, thus, strongly destabilized. This maximizes the energy difference between the two states and results in a conduction-bandwidth exceeding 400 meV (see Figure 3a). Conversely, in the case of the valence band the lobes of the wave functions are shifted such that both the Γ- and Z-point state display a “mixed” character with lobes of the orbital on one pentacene forming bonding as well as antibonding linear combinations with lobes on the neighboring pentacene (four white and three green arrows in Figure 3a for the Γ point and the inverse situation for the Z-point). As a consequence, the hybridization-induced stabilization/destabilization at the Γ- and Z-points is similar, resulting in a comparably small bandwidth and transfer integral (see Figure 3a,b). The reason for the difference between the VB and the CB is the different number nodes of the HOMO (four nodes) and LUMO (six nodes) along the long molecular axis, which results in a different spacing between the lobes (see Supporting Information and ref (41)). As detailed above, changing the stacking distance shifts neighboring pentacene units relative to each other, which concomitantly shifts the positions of the lobes of the respective orbitals. Thus, the pattern of bonding and antibonding hybridizations between molecular orbitals very much depends on the π-slip. Changing that slip can switch between situations with energetically very different or very similar Γ- and Z-point states. This is illustrated in the Supporting Information for a stacking distance of 7.08 Å, where for the valence band one observes a fully antibonding (bonding) state at the Γ- (Z-)point and, thus, a maximized transfer integral (in sharp contrast to the situation in Figure 3a calculated for a_3_ = 5.78 Å). These slip-dependent changes in orbital hybridizations then cause the variations of the bandwidths as a function of the stacking distance in Figure 2b.

**Figure 3:**
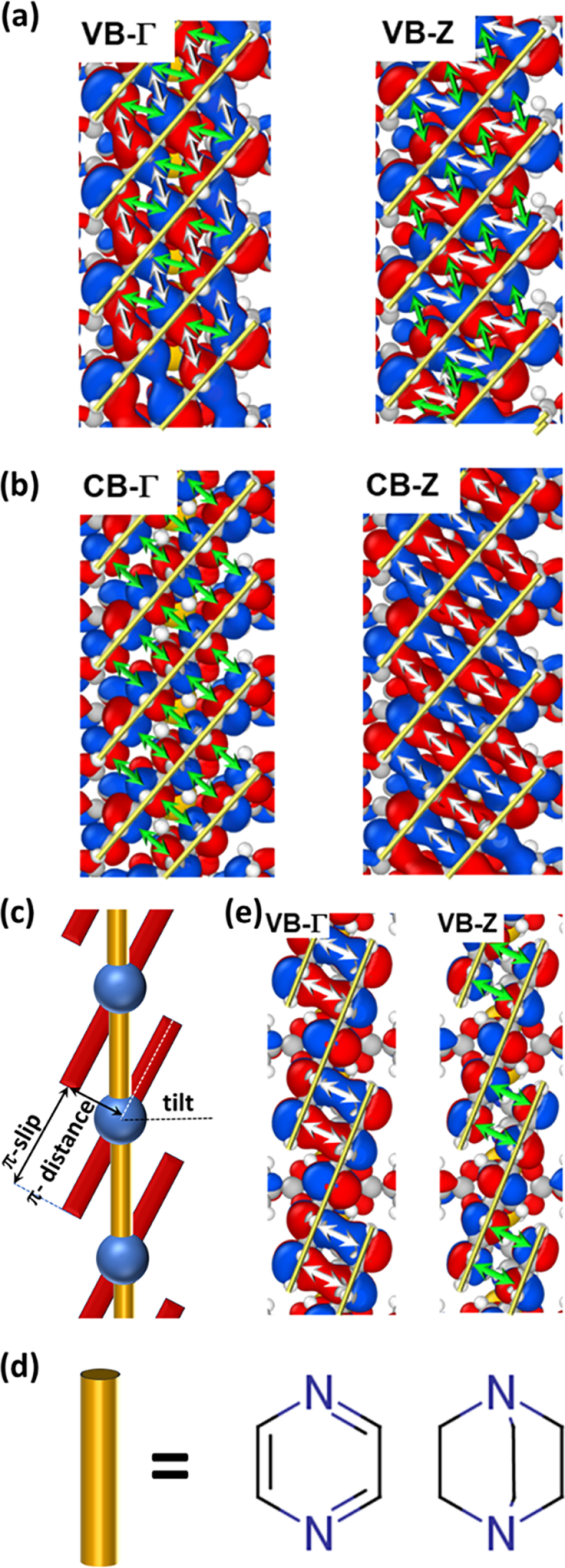
(a), (b): Isovalue plots of the electronic states at the Γ and at the Z points for the valence (a) and conduction band (b) in one of the pentacene stacks of the “paddle” structure. The white and green arrows denote bonding and antibonding hybridizations between π-lobes on neighboring pentacenes. The yellow lines highlight the location of the pentacene backbones. Panel (c) illustrates the concept of tuning the stacking distance by introducing suitable apical linkers represented by golden rods, with two examples of particular interest for the present family of systems shown in panel (d). Finally, panel (e) shows isovalue plots of the electronic states at the Γ and the Z points of the valence band of the “DABCO” structure.

The different periods of the variations of the widths of the valence and conduction bands in Figure 2b eventually cause a rather complex evolution of the band gap upon modifying the stacking distance, as shown in the Supporting Information.

The observation that conformations with larger stacking distances are characterized by particularly large widths of the valence band raises the question, whether this could be exploited for designing structures with improved hole-transport properties. Here the undercoordinated nature of the Zn^2+^ ions in the paddlewheels comes into play, as these ions readily bond to N-containing moieties, which could be used to introduce spacers that control the distance between the paddlewheels (see Figure 3c).^61,62^ Ideally, suited for our purpose would be, for example, pyracine and 1,4-diazabicyclo[2.2.2]octane (DABCO), which when used as apical linkers result in calculated a_3_ values of 9.53 and 9.47 Å, respectively. According to the data in Figure 2a,b, this puts such systems in the immediate vicinity of a local maximum of the electronic coupling for the valence band. Indeed, the calculated bandwidths and transfer integrals for these systems are rather large, and also the conduction-band related parameters are close to a local maximum. Notably, especially DABCO pillars have been repeatedly used^63,64^ employing growth techniques analogous to those employed for the pentacene MOFs from ref (34). This makes them highly promising for an experimental realization of the suggested tuning approach. The origin of the large transfer integral for the “DABCO” system is illustrated in Figure 3e, where we show that the increased slip due to the larger stacking distance causes a fully bonding coupling at the Γ point and a fully antibonding coupling at the Z point for the valence band. A disadvantage of the larger slip is, however, that it reduces the geometric overlap between neighboring pentacenes, which puts a certain limit to the achievable electronic coupling (compared, e.g., to the conduction band in the “paddle” case).

Nevertheless, conformations with large a_3_ values are highly interesting for maximizing charge transport, as the benefit from the fact that the charge-carrier mobility scales (approximately) quadratically with the distance between the centers of neighboring pentacene units (and thus with a_3_). To illustrate that, Figure 4a shows the evolution of the effective mass, m*, for electrons and holes (calculated from the band curvatures along ΓZ at the respective band extrema) as a function of the stacking distance. For band transport within the Drude model, m* relates to the charge-carrier mobility, μ_D_, as^65^
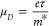
1Here, e is the elementary charge and τ represents the scattering time of the electrons, which is determined by both static and dynamic disorder with the latter being primarily determined by lattice phonon modes (see introductory paragraphs). For purely cosine-shaped bands within the tight binding model and applying the standard definition of the effective mass,^65^ m* becomes inversely proportional to the transfer integral, t, and to the square of the unit-cell dimension, a_3_^2^.

**Figure 4:**
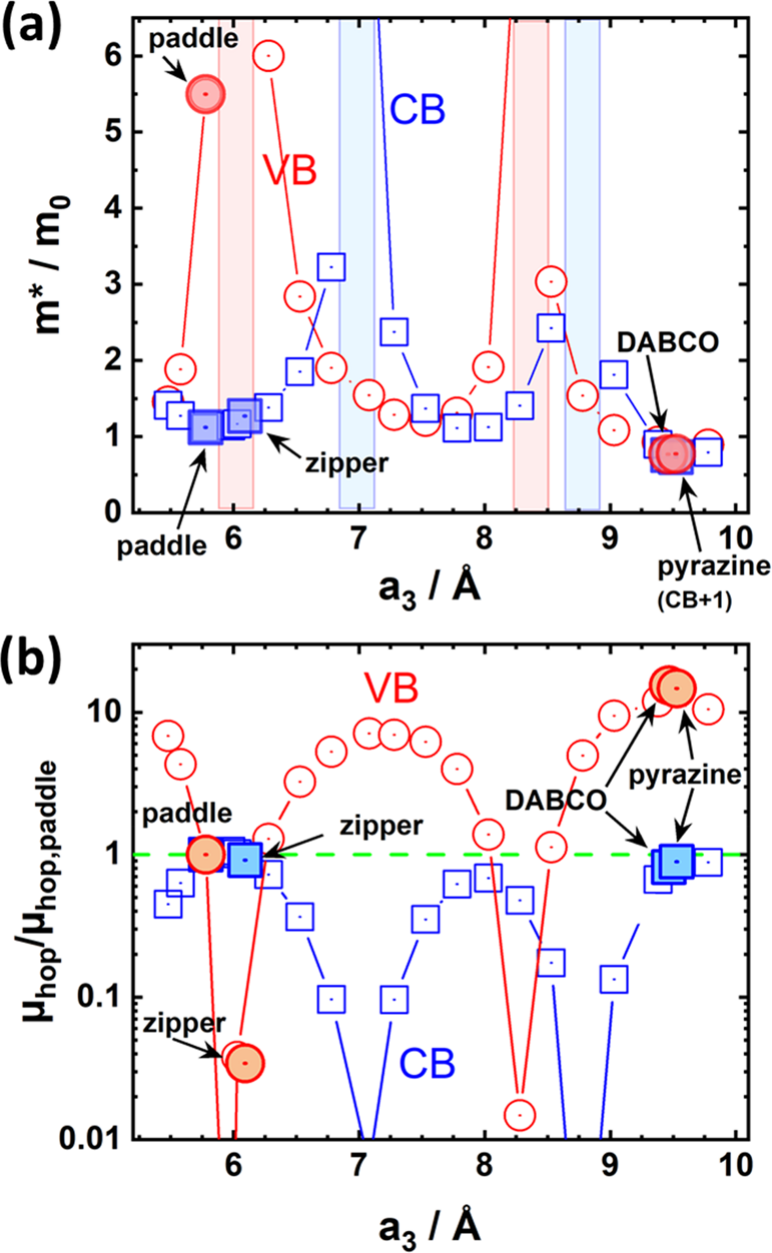
Evolution of the effective masses (a) and relative hopping mobility (b) defined in the main text for the valence (red) and conduction bands (blue) along ΓZ and the parallel **a**_**3**_ direction upon varying the stacking distance a_3_ in the model system. The values for the “paddle”, “zipper”, “pyrazine”, and “DABCO” structures are also shown as larger, shaded symbols. Again, they align along the evolution of the model system. The red and blue shaded areas in panel a highlight regions around values of a_3_ at which the respective effective masses diverge. The reason, why the relative hopping mobility is typically much larger for the VB than for the CB is the much smaller value of the respective hopping mobility μ_hop,paddle_ as the normalizing factor in the denominator.

Concerning the other limiting transport mechanism, pure hopping transport, the hopping mobility is given by^34^
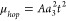
2with the proportionality constant A determined by the sample temperature and the reorganization energy. Assuming that the reorganization energy is not massively influenced by the stacking distance, which is the case at least for the internal reorganization energy in the strong coupling regime, a useful quantity to plot would be the ratio of the hopping mobility of a specific structure to that of the parent (paddlewheel) system, μ_hop_/μ_hop,paddle_. This ratio is shown in Figure 4b.

The displayed trends generate a clear picture (with key parameters from PBE and HSE06 simulations summarized in the Supporting Information): compared to the parent paddlewheel system from ref (34), introducing a pyrazine or DABCO linker between the paddlewheels decreases the effective mass for hole transport along the pentacene stacking direction by a factor of 7.2. The effect is even more pronounced for the hopping mobility of holes, which increases by a factor of 15. With 31 and 450 the respective ratios are even much larger, when referenced to the zipper case. For electron transport, the impact of the spacers is comparably weak with similar effective masses and estimates for the hopping mobilities as in the “paddle” system. This is simply a consequence of a rather large transfer integral for electrons already in the original “paddle” case, but here one has to keep in mind that the electron affinities of pentacene-based systems are typically not large enough for allowing electron injection. Thus, what really counts is the expected improvement of the mobility of holes by approximately an order of magnitude.

This being said, one should not forget that the data in Figure 4 do not account for the possibility that changing the MOF structure could also change lattice phonons and their impact on transport (even impacting the transport mechanism). Explicitly calculating the contribution of such vibrations goes beyond the scope of this paper. We, however, expect the impact of dynamic disorder not to increase (and potentially even to decrease) for a more suited stacking distance. This is because when starting from a conformation with a small electronic coupling, minor thermally induced variations of the slips might easily create a situation with a vanishing transfer integral (see Figure 2a,b). This has been repeatedly observed in the dynamic simulations in ref (34), and it results in a (dynamical) blocking of charge transport along the essentially 1D pentacene stacks. To encounter such a vanishing value of the transfer integral in the “DABCO” or “pyrazine” cases appears much more unlikely, as it would require much larger changes in the local geometry (see Figure 2a,b).

In summary, it is rather well established in the organic semiconductor community that changing the relative alignment of π-conjugated backbones has a profound impact on the electronic coupling between the π-systems and, thus, on transport properties. The pentacene-based MOF first described in ref (34) represents an intriguing example for a system in which this arrangement could be tuned through an extrinsic framework structure. To exploit the potential of such an approach, we here provide guidelines for how the electronic coupling between occupied frontier states in such a system can be massively increased through structural design. This is expected to boost the hole mobility by one (or several) order(s) of magnitude (depending on whether the original structure is actually closer to the “paddle” or “zipper” conformation). Most importantly, from a practical point of view, bearing in mind the undercoordinated nature of the Zn paddlewheels and the chemical nature of the suggested spacers, we expect the necessary modifications to be relatively straightforward to implement experimentally, which allows the suggested materials to realize the full potential of through-space charge transport in MOFs. For the organic semiconductor community, the present paper provides a showcase for how framework design can be used to realize structures hitherto not accessible in molecular crystals.

## Supporting Information Available

Additional information on the employed methodology, lattice parameters, survey plots of band structures, a discussion of the interstack coupling of electronic states, detailed geometrical data, the dependence of the transfer integral on distance for a cofacial pentacene dimer, the evolution of the bandwidth with a3, G- and Z-point states of the valence band for a_3_ = 7.08 Å, tests of the impact of the choice of the functional, additional information on polymorph properties, and an in-depth discussion of the tight-binding fits (PDF)
