# Supplementary material for: Maximizing the Carrier Mobilities of Metal–Organic Frameworks Comprising Stacked Pentacene Units
Source: J Phys Chem Lett. 2021 Jul 20;12(29):7002–9. doi: 10.1021/acs.jpclett.1c01892 (PMC8397338; doi:10.1021/acs.jpclett.1c01892)
Supplement: Supplementary file 1 — jz1c01892_si_001.pdf [file jz1c01892_si_001.pdf]

Supporting Information for

Maximizing the Carrier Mobilities of Metal-Organic Frameworks Comprising Stacked Pentacene Units

Egbert Zojer and Christian Winkler*

Institute of Solid State Physics, Graz University of Technology, NAWI Graz, Petersgasse 16, 8010 Graz, Austria

Corresponding Author

* Email: Egbert.zojer@tugraz.at

The input and output files of all calculations reported in this manuscript can be found in the NOMAD database using the following DOI: 10.17172/NOMAD/2021.07.08-1

Additional information on the employed methodology

For our studies we used the FHI-aims code [1–4] employing periodic boundary conditions. Following the results of convergence tests, we chose a 2×2×7 k-points grid. We primarily used the Perdew-Burke-Ernzerhof (PBE) functional [5,6], which was coupled to a revised variant of the many-body dispersion correction.[7] For the basis-set we employed the FHI-aims default settings, which are discussed in more detail below. Convergence criteria for the self-consistency cycle were set to the default values for changes in the charge density (10^{-5}), the total energy (10^{-6} eV), the forces (10^{-4} eV·Å^{-1}). To determine the occupation of the Kohn-Sham eigenstates a Gaussian broadening function with a width of σ = 0.01 eV was used. As the studied system contains Zn atoms, the atomic ZORA approximation was used [8] to account for relativistic effects.

As generalized gradient functionals like PBE severely underestimate band gaps and also often produce somewhat too small band widths, the electronic structure of selected systems was recalculated using the hybrid Heyd-Scuseria-Ernzerhof (HSE06) exchange correlation functional [9] with a screening parameter of omega=0.11 Bohr^{-1}.[10]

Local geometry optimizations were performed using the version of the Broyden-Fletcher-Shanno-Goldfarb optimization algorithm enhanced by the trust radius method [1], with a tolerance threshold of 10^{-2} eV·Å^{-1}. For the main systems ("paddle", "zipper", "distorted", "pyrazine", and "DABCO"), a unit-cell relaxation was performed simultaneously optimizing the unit-cell lengths and the atomic positions, but fixing the unit-cell vectors orthogonal to each other (i.e., enforcing an orthorhombic unit cell). For systems with artificially modified a_3 lattice constants, only the atomic positions were relaxed.
Plots of the MOF geometries were produced using OVITO [11]. Marvinsketch was used for drawing, chemical structures, ChemAxon (https://www.chemaxon.com)

Employed basis sets

The basis functions employed in the FHI-aims simulations have the format

$$\Phi(r) = \frac{u(r)}{r} * Y_{lm}(\Theta, \Phi)$$

in spherical coordinates (r, Θ, Φ) relative to a given atomic center. FHI-aims provides for every atomic species a preconstructed *species_defaults* file. The used tight basis sets were not further adjusted.

Table S1. Basis functions that have been used for all calculations performed with FHI-aims[12]. The abbreviations read as follows[1]: X(nl, z), where X describes the type of basis function where H stands for hydrogen-like functions and ionic for a free-ion like radial function. The parameter n stands for the main/radial quantum number, l denotes the angular momentum quantum number (s, p, d, f, …), and z denotes an effective nuclear charge, which scales the radial function in the defining Coulomb potential for the hydrogen-like function. In the case of free-ion like radial functions, z specifies the onset radius of the confining potential. If auto is specified instead of a numerical value, the default onset is used.

	H	C	N	O	Zn
Minimal	valence (1s, 1.0)	valence (2s, 2.0)	valence (2s, 2.0)	valence (2s, 2.0)	valence (4s, 2.0)
	ion_occ (1s, 0.5)	valence (2p, 2.0)	valence (2p, 3.0)	valence (2p, 4.0)	valence (3p, 6.0)
		ion_occ (2s, 2.0)	ion_occ (2s, 1.0)	ion_occ (2s, 1.0)	valence (3d, 10.0)
		ion_occ (2p, 2.0)	ion_occ (2p, 2.0)	ion_occ (2p, 3.0)	ion_occ (4s, 1.0)
					ion_occ (3p, 6.0)
					ion_occ (3d, 9.0)
First tier	H(2s, 2.1)	H(2p, 1.7)	H(2p, 1.8)	H(2p, 1.8)	H(2p, 1.7)
	H(2p, 3.5)	H(3d, 6)	H(3d, 6.8)	H(3d, 7.6)	H(3s, 2.9)
		H(2s, 4.9)	H(3s, 5.8)	H(3s, 6.4)	H(4p, 5.4)
					H(4f, 7.8)
					H(3d, 4.5)
Second tier	H(1s, 0.85)	H(4f, 9.8)	H(4f, 10.8)	H(4f, 11.6)	
	H(2p, 3.7)	H(3p, 5.2)	H(3p, 5.8)	H(3p, 6.2)	
	H(2s, 1.2)	H(3s, 4.3)	H (1s, 0.8)	H (3d, 5.6)	
	H(3d, 7.0)	H(5g, 14.4)	H(5g, 16)	H(5g, 17.5)	
		H(3d, 6.2)	H(3d, 4.9)	H(1s, 0.75)	

Additional data

[1] As described in the FHI-aims manual, version January 23, 2017.

Summary of the calculated key transport parameters

Table S2: PBE and HSE calculated effective masses and relative hopping mobilities for the “paddle”, “zipper”, “pyrazine”, and “DABCO” structures. For the structures containing the spacers suggested in this work, also the ratio between the effective mass of the parent “paddle” system and the effective mass for the “pyrazine” and “DABCO” structures is provided. The HSE06 values have been included to show that the discussed trends are hardly impacted by the employed functional.

		VB		CB	
		PBE	HSE06	PBE	HSE06
paddle	m^*/m_0	5.50[§]	4.73	1.11[§]	0.98
zipper	m^*/m_0	23.7	18.7	1.26	1.03
pyrazine	m^*/m_0	0.77	0.66	0.72[&]	0.62[&]
	m^*_{paddle}/m^*	**7.2**	**7.2**	1.5	1.5
	$\mu_h/\mu_{h,paddle}$	14.7	14.8	0.89	0.92
DABCO	m^*/m_0	0.76	0.66	0.75	0.65
	m^*_{paddle}/m^*	**7.2**	**7.1**	1.5	1.6
	$\mu_h/\mu_{h,paddle}$	15.4	15.6	0.81	0.84

[&] these values have been obtained for the CB+1, as the states associated with the lowest unoccupied band in the pyrazine-containing MOF are localized on the pyrazines.

[§] these values are somewhat smaller than those reported in the Supporting Information of [13]*, which we primarily attribute to the different used vdW correction, which somewhat impacts the equilibrium distance and, thus, the slip for a specific value of* a_3 *(see below). Additionally, we here report m* parallel to* $\mathbf{a_3}$ *at the* Γ*-point, while in* [13] *a thermal averaging has been performed.*

Table S3. Comparison between the PBE and HSE06 calculated transfer integrals and bandwidths for the “paddle”, “pyrazine”, and “DABCO” structures. The four rightmost columns show the ratios between the values for “pyrazine” or ”DABCO” structures and the parent “paddle” system.

	paddle:		pyrazine:		DABCO:		pyrazine/paddle		DABCO/paddle	
	t/meV	BW/eV	t/meV	BW/eV	t/meV	BW/eV	t_{py}/t_{pa}	BW_{py}/BW_{pa}	t_{DA}/t_{pa}	BW_{DA}/BW_{pa}
VB										
PBE	-27.1	0.11	-63.0	0.25	-65.0	0.26	2.33	2.35	2.40	2.43
HSE06	-31.2	0.12	-73.0	0.29	-75.4	0.30	2.34	2.37	2.41	2.44

CB			for CB+1							
PBE	104.8	0.42	60.0	0.24	57.8	0.23	0.57	0.57	0.55	0.55
HSE06	121.4	0.49	70.4	0.28	68.1	0.27	0.58	0.58	0.56	0.56

Lattice parameters of all fully optimized structures

Table S4: Lengths of the unit cell vectors for the different structures and polymorphs. Please note that all unit cells were forced to be orthorhombic. "Paddle" refers to the original structure proposed in [14], "zipper" to the lowest energy conformation in which linkers in $\mathbf{a}_1$ and $\mathbf{a}_2$ directions connect two different Zn atoms such that no paddlewheels are formed, "distorted" refers to the structure with distorted linkers in $\mathbf{a}_2$ direction and tilted paddlewheels (see below). "pyrazine" and "DABCO" designate the structures with pyrazine and DABCO molecules acting as spacers and connecting neighboring paddlewheels (see Figure 4 c and d).

structure/polymorph	a_1 / Å	a_2 / Å	a_3 / Å
"paddle"	19.453	19.453	5.780
"zipper"	18.924	18.922	6.093
"distorted"	19.433	19.340	5.606
"pyrazine"	19.772	19.693	9.528
"DABCO"	19.715	19.715	9.466

Frontier band structures

Figure S1 contains the relevant band structures of all reported systems. additionally the conduction bands (top panels) and an even larger number of bands (bottom panels). Many of the bands appear in nearly degenerate pairs, which is a consequence of the two pentacene stacks per unit cell. This near degeneracy will be discussed in more detail in the next section. For the "pyrazine" structure one also sees entirely flat bands (including the CB). These correspond to states fully localized on the pyrazines.

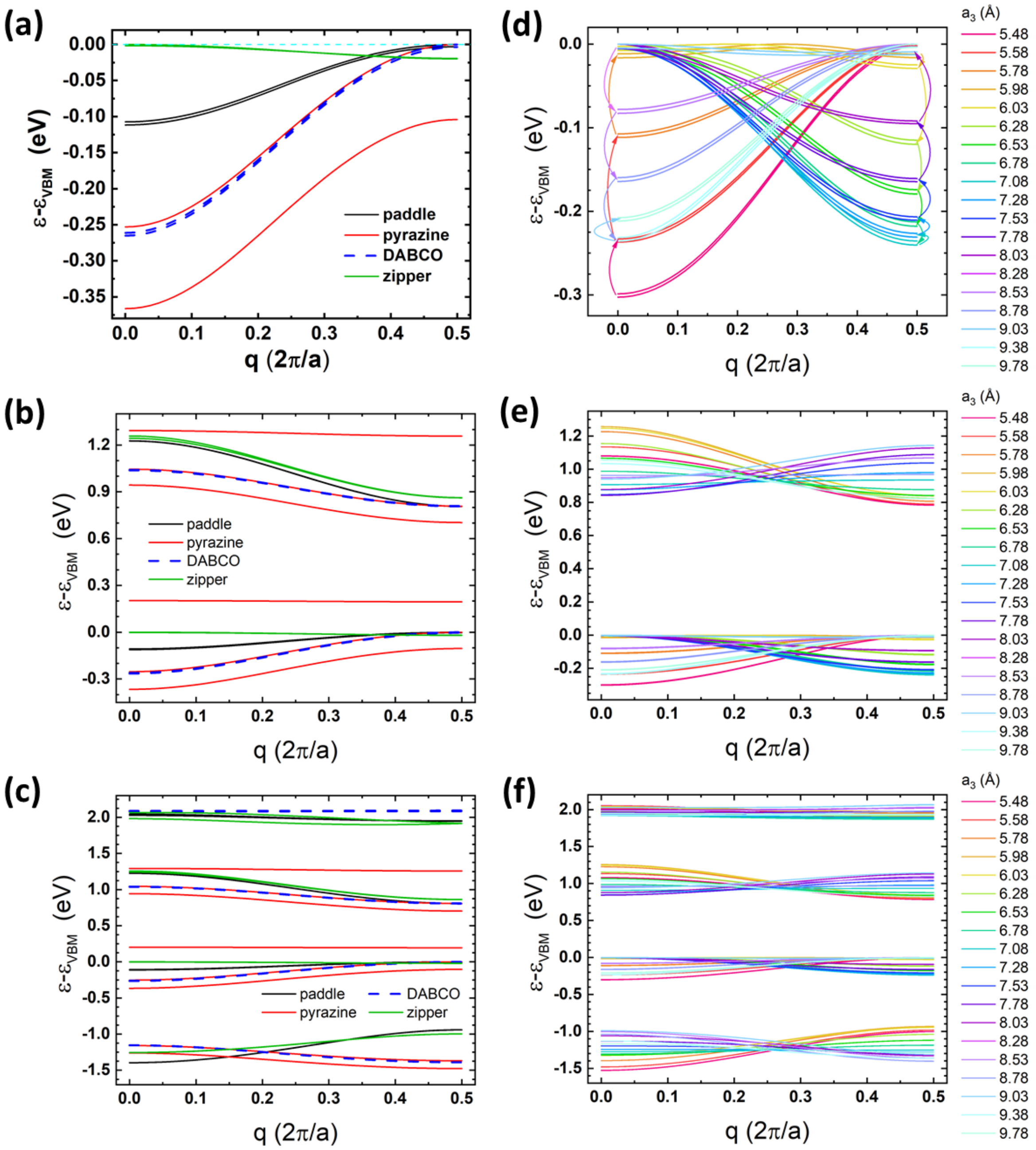

Figure S1. (a), Valence bands, (b) frontier bands, and (c) bands in the energetic vicinity of the frontier bands for the "paddle", "pyrazine", and "DABCO" structures of the pentacene-MOF in ΓZ. The three panels differ in the displayed energy window; (d), (e), (f) equivalent bands for the model systems as a function of the pentacene stacking distance.

Near degeneracy of the frontier bands

Figure S2 shows top views of isovalue plots of the Γ-point states of the frontier bands of the "paddle" parent system to illustrate the phase shifts between states in the four pentacene columns surrounding

a pore. In panel (b) it is indicated, whether the electronic coupling between neighboring columns is bonding (white arrows) or antibonding (green arrows). The energetic splitting between the two highest occupied bands amounts to ~4 meV, while that for the two lowest unoccupied bands is negligible. This can be understood from the same number of bonding and antibonding interactions in case of the CB and CB+1, while for the VB all interactions are antibonding and for the VB-1 they are all bonding.

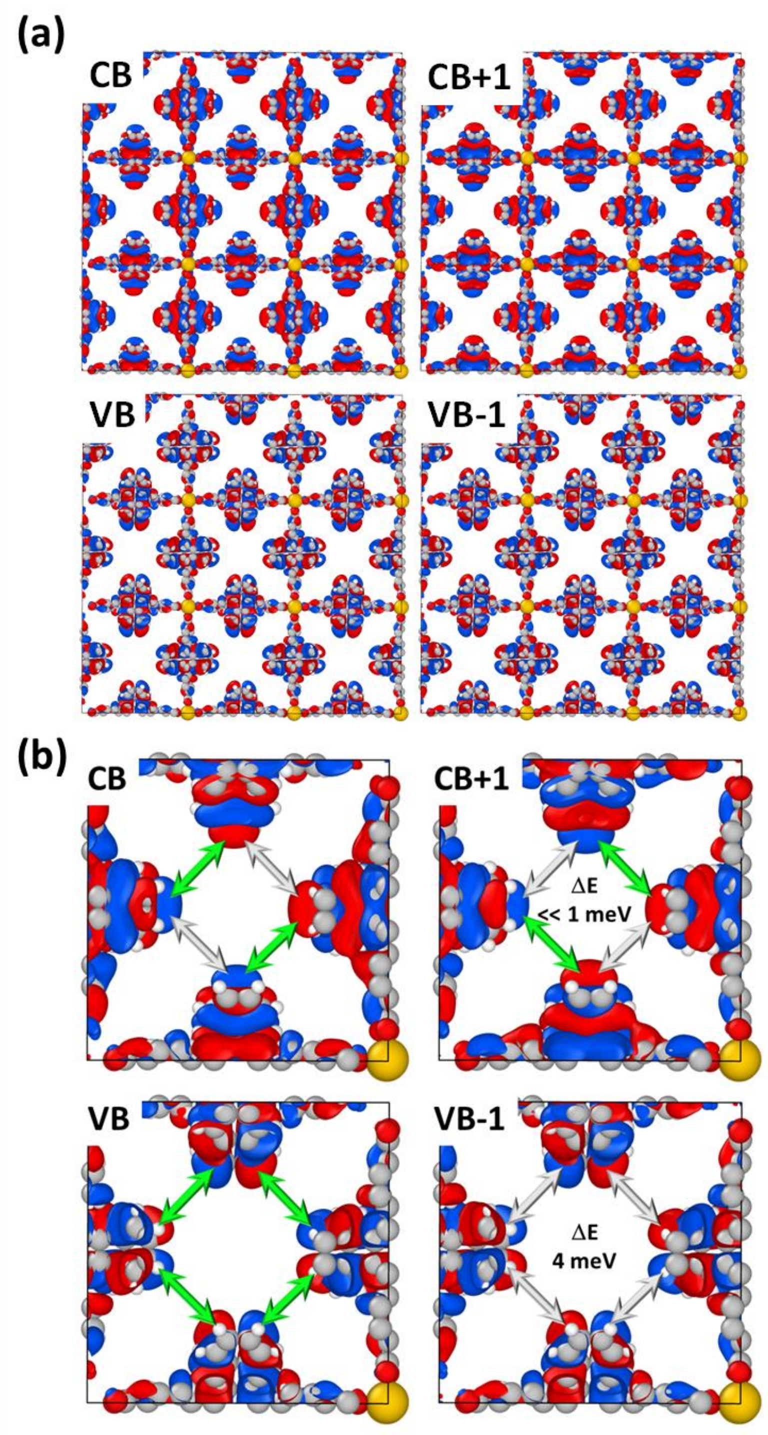

Figure S2. Top views of isovalue plots of the Γ-point states of the frontier bands of the "paddle" parent system. Panel (b) shows the zoom into an individual pore. The green and white arrows denote

antibonding and bonding couplings between the π-lobes on neighboring molecules. The values of ΔE are the splittings of the two frontier bands at the Γ-point.

Highest occupied and lowest unoccupied molecular orbitals of pentacene

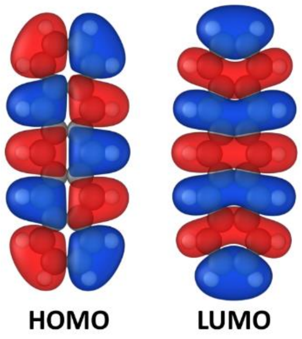

Figure S3. Isovalue plots of the highest occupied molecular orbital (HOMO) and lowest unoccupied molecular orbital (LUMO) of pentacene illustrating the different number of nodes of the woe wavefunctions.

Additional geometrical data

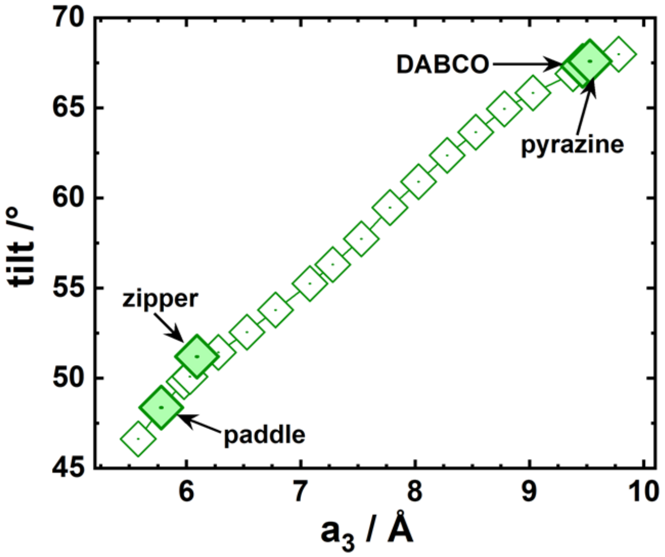

Figure S4: Tilt angle of the pentacene planes relative to the **a_1,a_2** plane as a function of the stacking distance (i.e., the value of a_3) for the model system (open symbols) and for the "paddle", "zipper", "DABCO", and "pyrazine" structures.

Dependence of the transfer integral on the dimer distance

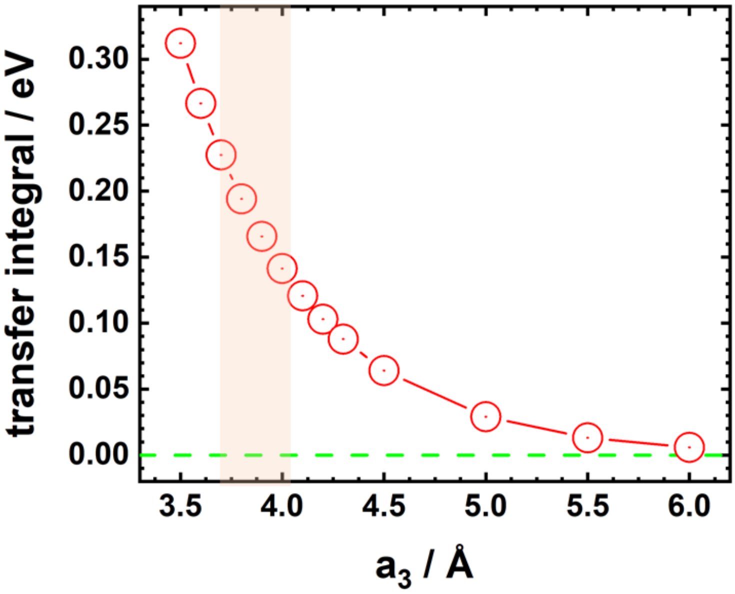

Figure S5: Dependence of the transfer integral for a cofacial pentacene dimer (calculated by the "energy splitting in dimers" approach[15] as 0.5 times the energetic splitting between the HOMO and the HOMO-1) as a function of the distance between the two molecules. The shaded region highlights the range of π-distances observed when varying the stacking distance. Here, it should, however, be noted that upon varying the stacking distance, the π-slip between the pentacene units changed as a function of the distance, which is not the case in the dimer considered in the current plot.

Dependence of the band gap on the stacking distance

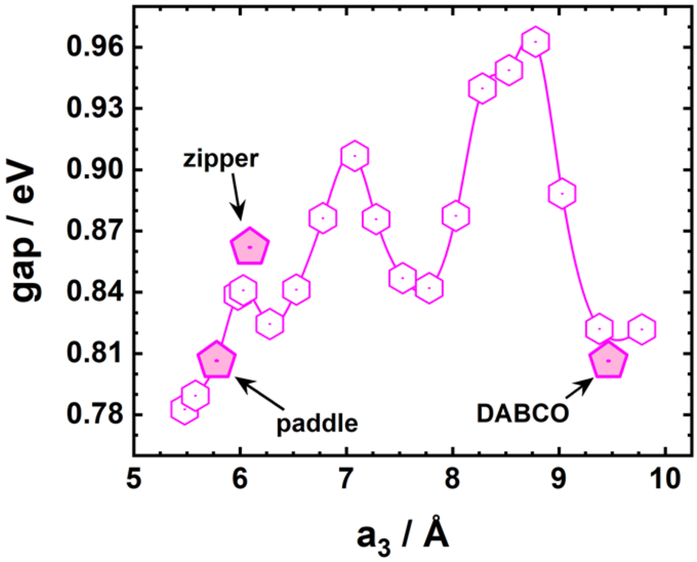

Figure S6: Dependence of the band gap of the studied MOF systems on the stacking distance. The MOF containing the pyrazine spacer is not considered here, as in that system the conduction band is

localized on the pyrazine units. The displayed evolution appears rather complex. This is a consequence of the fact that the periods in the evolutions of the widths of the valence and conduction bands are different (see main text). In a simple single-particle picture like the one applied here (i.e., disregarding polarization and excitonic effects) the band gap is primarily reduced when the valence and conduction bands simultaneously display a large dispersion. The overall small value of the gap is a consequence of the many-electron self-interaction error not mitigated in generalized gradient functionals.

Electronic states of the valence band for a stacking distance that maximizes the transfer integral

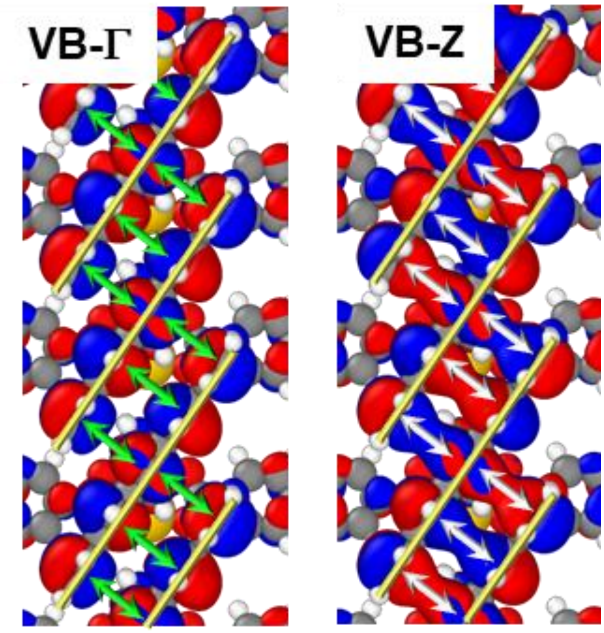

Figure S3: Isovalue plots of the electronic states at the Γ and at the Z points for the valence band in one of the pentacene stacks of the structure in which the stacking distance has been artificially increased to 7.08 Å. The white and green arrows denote bonding and antibonding hybridizations between π-lobes on neighboring pentacenes. The yellow lines highlight the location of the pentacene backbones. The plot reveals that for the chosen stacking distance the Γ-point state is fully antibonding, while the Z-point state is fully bonding such that the band width is maximized.

Methodology- and conformation-related tests

Comparison between PBE and HSE06 band structure

As expected, using the HSE06 hybrid functional instead of PBE results in somewhat larger band widths and a significantly increased energy gap between the valence and a conduction band. This, however does not at all affect the trends discussed in the current work, as shown in Table S2 and Table S3 included above.

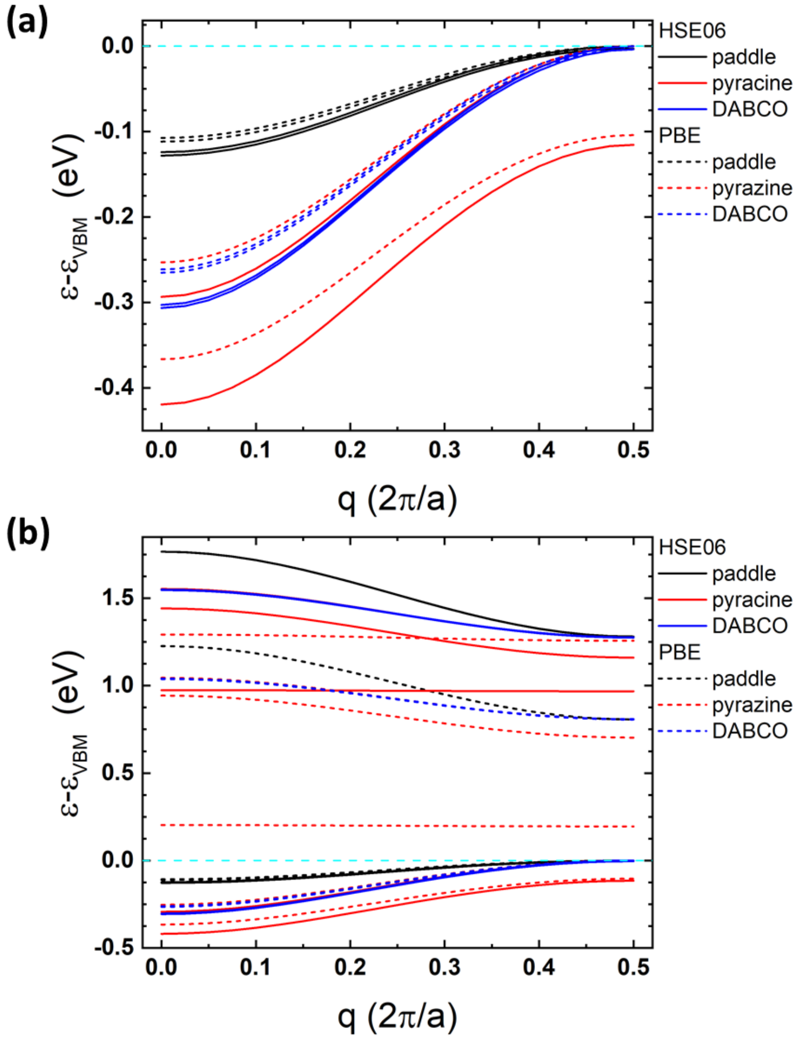

Figure S8. Comparison between the PBE and HSE06 calculated frontier bands of the "paddle", "pyrazine", and "DABCO" structures of the MOFs in ΓZ direction, with (a) showing a zoom into the valence band region.

Polymorph structures of the pentacene containing MOFs

As described in the main manuscript, several polymorphs were found for the structure without spacers in the course of the geometry optimizations. These comprise the paddlewheel structure (best consistent with the experimental data) and the zipper structure shown in Figures 1d and 1e in the main manuscript. Additionally, we observed a structure in which the linkers in the $\mathbf{a_1}$ direction were seriously distorted, as shown in Figure S9, which, however, resulted in a too small lattice parameter a_3 (of 5.61 Å) compared to the experiments (a_3=5.8 Å).

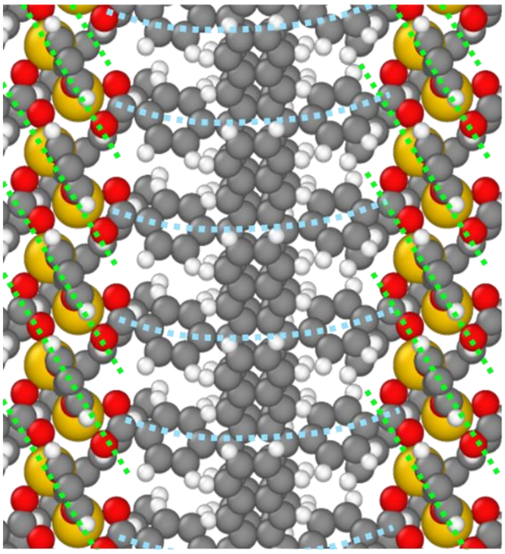

Figure S9. Geometry of the bent linkers of the "distorted" polymorph aligned along $\mathbf{a_1}$ direction. The massively tilted nodes are highlighted by the dotted green lines.

Notably, despite the worse agreement with experiments regarding lattice parameter a_3, both the "distorted" and "zipper" structures came out lower in energy than the parent "paddle" polymorph, as shown in Figure S10.

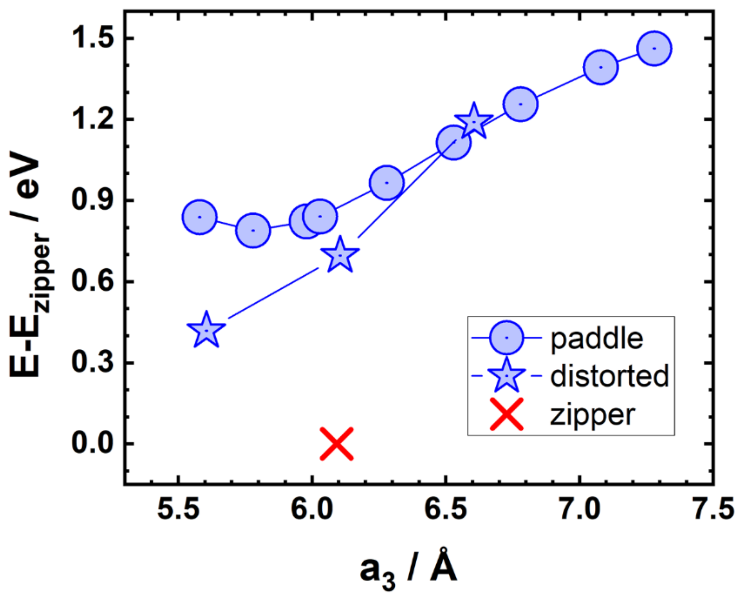

Figure S10. Total energy of the "paddle", "distorted" and "zipper" polymorphs as a function of lattice parameter a_3. For the lowest energy structures of each polymorph also the lattice parameters a_1, a_2, and a_3 were optimized (but keeping the angles between the lattice vectors fixed at 90°). The "distorted" polymorph was not stable for larger values of a_3 and considering that no individual paddlewheels exist in the "zipper" structure, for that polymorph only the data point of the equilibrium conformation is shown (which is the lowest energy structure we observed in all our calculations).

Impact of the used van der Waals correction and the polymorph on the structural parameters of the MOF

Figure S11 shows that especially the slip of neighboring π-planes and their tilt relative to the $\mathbf{a_1,a_2}$ plane are very similar for the "paddle" and "distorted" polymorphs. The same applies to two selected "paddle" type structures for which the atomic positions were optimized using the TS[16] instead of the MBD-NL[7] van der Waals correction. As far as the normal distances of neighboring π-planes are concerned, they are somewhat larger in the "distorted" pentacene stacks and smaller in the TS-optimized ones, where the deviations from the regular "paddle" structures are typically only on the order of 0.1 Å. Only for the fully optimized "distorted" structure the increase in distance is somewhat larger (~0.25 Å).

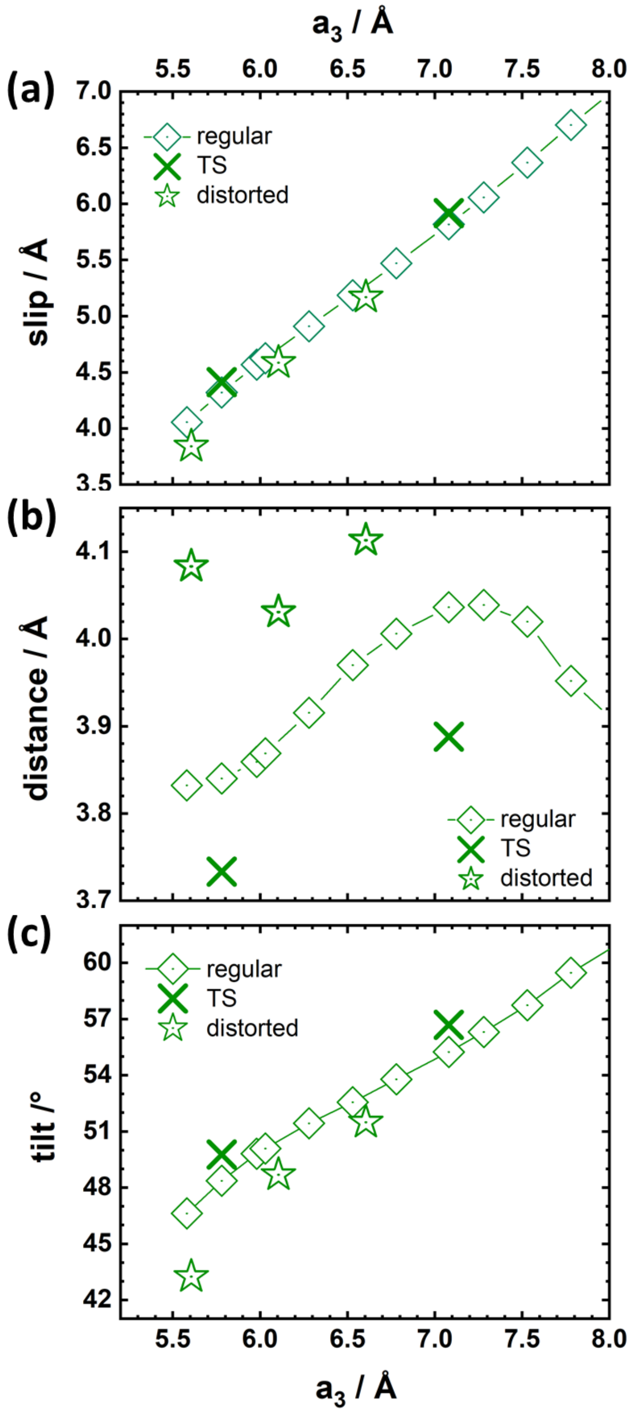

Figure S11. Comparison of a_3-dependence of the slip of neighboring π-planes (a) and their π-distance (b), as well as the tilt angle of the pentacene units relative to the $\mathbf{a}_1$, $\mathbf{a}_2$ plane for the regular structure derived from the "paddle" polymorph and optimized with the MBD-NL van der Waals correction (regular), for the "distorted" polymorph also optimized with the MBD-NL correction, and for two "paddle" structures (for the a_3 values of the MBD-NL optimized equilibrium structure and for an a_3 value close to the maximum of the valence band widths) calculated employing the TS van der Waals correction. In the TS calculations, only the atomic positions have been optimized.

Figure S12 shows the impact of the modified geometries discussed above on the band widths and effective masses, where one sees that the “distorted” structures as well as the TS-optimized structures align well the original evolution, with somewhat larger deviations for the effective mass close to its divergence in the TS case. This is not really surprising considering that this parameter close to a zero-crossing of the band width displays an extreme dependence on the exact value of the slip and, thus, on how close the chosen geometry lies to the actual zero-crossing of the band with (i.e., the flat-band case).

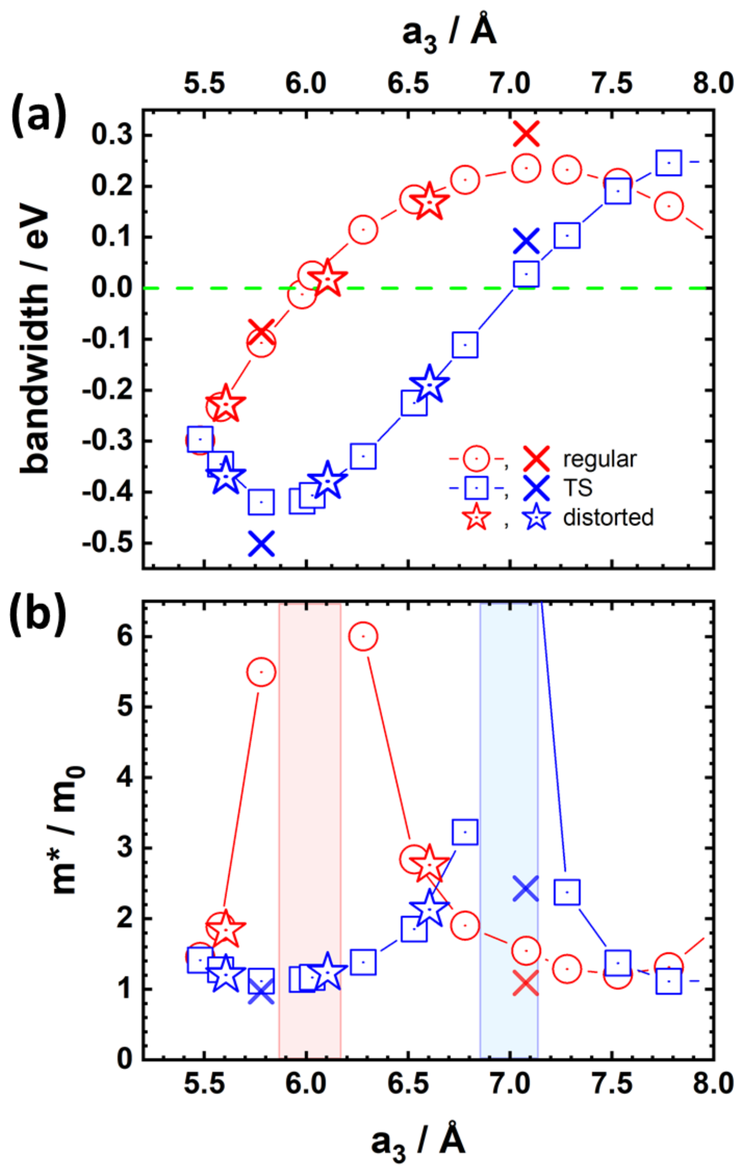

Figure S12. Comparison of the evolution of the band width and effective mass for the valence (red) and conduction bands (blue) calculated for the model structure discussed in the main manuscript, for the “distorted” polymorph, and for the structures optimized with the TS correction discussed in detail in the previous paragraphs and figures.

Details on the tight-binding fits used to determine the transfer integrals

The general form of the tight-binding band structure for band I with one pentacene unit in the unit cell is:

$$\varepsilon_i(\boldsymbol{k}) = \varepsilon_{i,0} + \sum_{\boldsymbol{R_j}} t_{i,R_j} e^{i\boldsymbol{kR_j}}$$

Here, $\varepsilon_{i,0}$ corresponds to the center of band i, the sum runs over the considered (nearest) neighbors and the t_{i,R_j} are the corresponding transfer integrals. This function is fitted to the DFT calculated band structure either for a self-consistent dense sampling of the entire 1. BZ (with 4×4×14 k-points), when fitting a 3D tight binding model, or to the band running in ΓZ direction for the 1D model considering only the electronic coupling between pentacene molecules within one pentacene stack.[17] The latter has been done for determining all transfer integrals contained in the main manuscript, where only $\boldsymbol{R_j}$ equaling $\mathbf{a_3}$ and $\mathbf{-a_3}$ has been considered, forcing the corresponding two transfer integrals to be equal for symmetry reasons. The latter implies a cosine-type shape of the bands. This ansatz works very well for all bands with appreciable band dispersion, as will be shown for selected cases in the following. Subsequently, we will also discuss the impact of fitting a 3D tight binding model and show a few pathological cases (systems with very small band widths), in which also next nearest neighbor interactions need to be considered in order to properly reproduce the very weakly dispersing bands.

Table S5. Transfer integrals for the main structures and polymorphs of the MOF fitted by 1D tight-binding models. The transfer integrals for **a_3** and **-a_3** as well as for **2a_3** and **-2a_3** were forced to be equal**.** For the two left columns only nearest neighbor transfer integrals were considered in the fits, while for the four rightmost columns also next-nearest neighbor transfer integrals were included as well.

model	**a_3, -a_3**		**a_3, -a_3, 2a_3, -2a_3**			
	t_{VB} / meV	t_{CB} / meV	t_{VB} / meV		t_{CB} / meV	
"paddle"	-27.1	104.8	-27.1	-0.89	104.8	0.77
"zipper"	4.8	95.2	4.8	-0.13	95.2	2.2
"pyracine"	-63.0	60.0	-63.0	-2.5	60.0	0.59
"DABCO"	-65.0	57.8	-65.0	-2.8	57.8	0.34

VB CB

"paddle"

a_3, -a_3

"paddle"

a_3, -a_3, 2a_3, -2a_3

"zipper"

a_3, -a_3

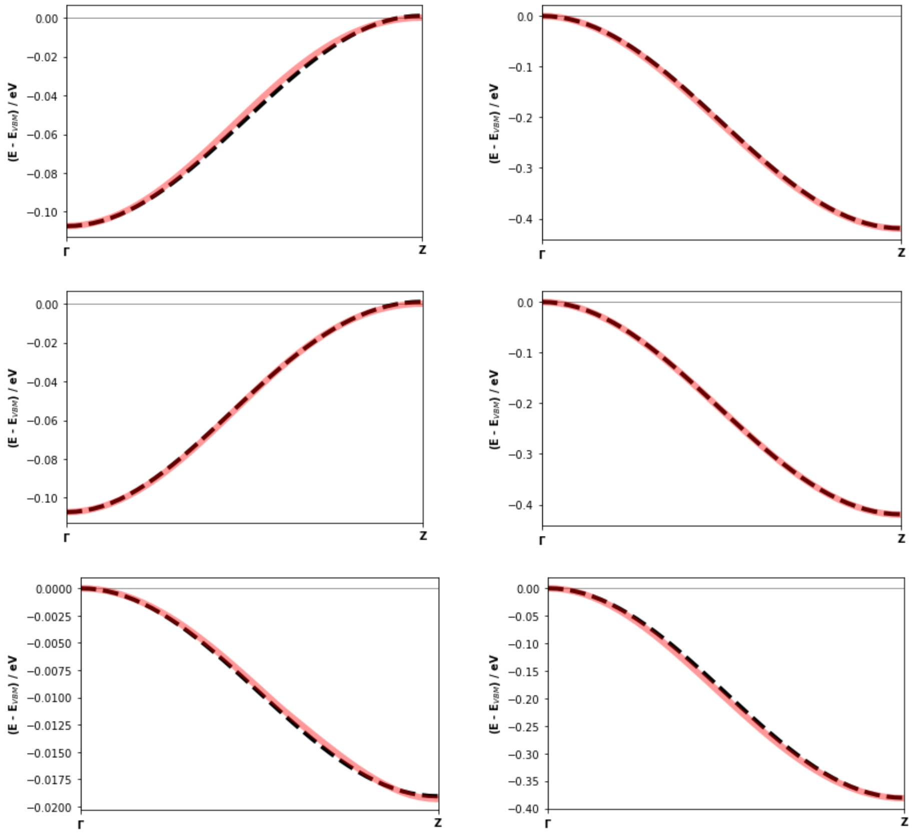

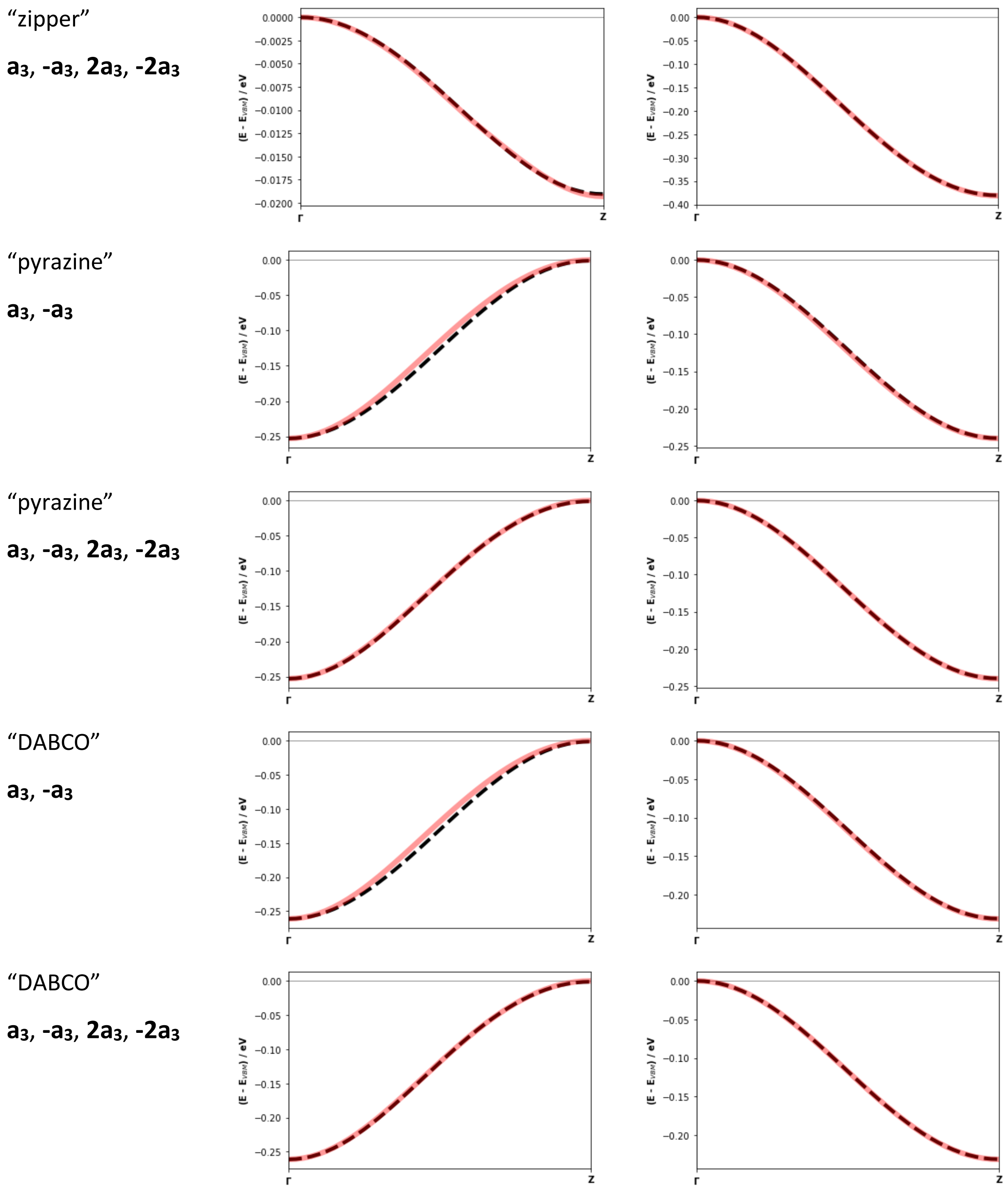

Figure S13. DFT calculated structures of the valence band (left) and the conduction band (right) for the "paddle", "zipper", "pyrazine" and "DABCO" conformations (solid red lines) and results of the 1D tight-binding fits (dashed black lines) using the ansatz described in the leftmost column.

The results contained in Table S5 show that for the systems considered there, 1D nearest neighbor tight-binding fits are sufficient with next-nearest neighbor values being above 1 meV only for the valence bands of the "pyrazine" and "DABCO" structures. This is consistent with the quality of the fits illustrated in Figure S13, where only for these two cases minor deviations between the DFT calculated

band structures and the tight-binding fits considering only nearest-neighbor interactions are observed. The data contained in Table S6 illustrate that the situation is very well described by the usually applied 1D tight binding models with transfer integrals in **a$_1$** and **a$_2$** directions being vanishingly small.

Table S6. Transfer integrals for the “paddle”, “zipper”, and “DABCO” systems obtained by fitting a 3D nearest neighbor tight-binding models. The transfer integrals for **a$_1$** and **-a$_1$** , **a$_2$** and **-a$_2$** , **a$_3$** and **-a$_3$** were forced to be equal.

	$t_{VB,a1}$ / meV	$t_{VB,a2}$ / meV	$t_{VB,a3}$ / meV	$t_{CB,a1}$ / meV	$t_{CB,a2}$ / meV	$t_{CB,a3}$ / meV
“paddle”	0.28	0.31	-27.2	0.12	0.13	104.7
“zipper”	-0.35	-0.35	4.5	-0.07	-0.24	96.2
“DABCO”	-0.03	-0.02	-65.0	-0.11	-0.10	57.8

Only in cases in which the transfer integrals become particularly small, the bands deviate from a cos-type shape and next-nearest neighbor transfer integrals become comparable to nearest neighbor ones due to a superexchange (i.e., nearest-neighbor mediated) type of coupling.[17] This is illustrated for the cases in which such problems occur in Table S6 and Figure S14, where one should note the extremely small energy scales in the panels in Figure S14. Thus, these more complex cases are of no relevance for the discussion in the main manuscript as they correspond to essentially vanishing band widths and transfer integrals.

Table S7. Transfer integrals for the structures considered in Figure S14 fitted by 1D tight-binding models. The transfer integrals for **a$_3$** and **-a$_3$** as well as for **2a$_3$** and **-2a$_3$** were forced to be equal. For the two left columns only nearest neighbor transfer integrals were considered in the fits, while for the four rightmost columns also next-nearest neighbor transfer integrals were included.

distance / band	**a$_3$, -a$_3$**	**a$_3$, -a$_3$, 2a$_3$, -2a$_3$**	
	t / meV	t / meV	
VB 5.98 Å	-0.32	-0.32	-2.9
VB 6.03 Å	5.1	5,1	3.1
VB 8.28 Å	2.3	2.3	0.96
CB 8.78 Å	1.3	1.3	0.67

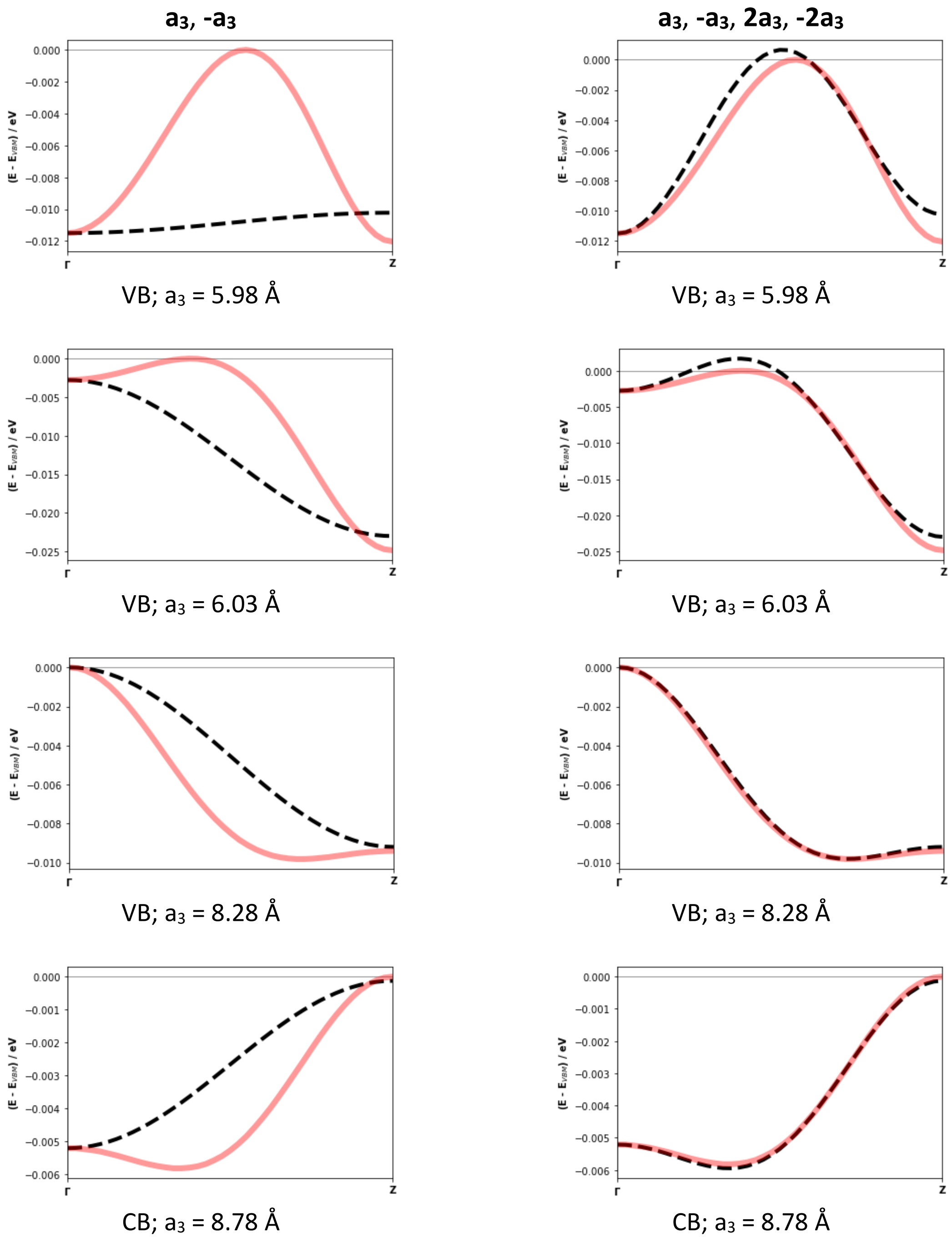

Figure S14: Examples for geometries at which the nearest neighbor transfer integrals become so small that the band structure deviates from a cosine-type shape. The left panels show the situation including only nearest-neighbor couplings, while in the right panels also next nearest neighbor couplings are considered. The structures are derived from the "paddle" structure with artificially increased unit-cell sizes in $\mathbf{a_3}$ direction (for details see main manuscript). The DFT calculated band structures are shown as solid red lines, while the dashed black lines denote the tight binding fits.

(1) Blum, V.; Gehrke, R.; Hanke, F.; Havu, P.; Havu, V.; Ren, X.; Reuter, K.; Scheffler, M. Ab Initio Molecular Simulations with Numeric Atom-Centered Orbitals. *Computer Physics Communications* **2009**, *180* (11), 2175–2196. https://doi.org/10.1016/j.cpc.2009.06.022.
(2) Havu, V.; Blum, V.; Havu, P.; Scheffler, M. Efficient O(N) Integration for All-Electron Electronic Structure Calculation Using Numeric Basis Functions. *Journal of Computational Physics* **2009**, *228* (22), 8367–8379. https://doi.org/10.1016/j.jcp.2009.08.008.
(3) Marek, A.; Blum, V.; Johanni, R.; Havu, V.; Lang, B.; Auckenthaler, T.; Heinecke, A.; Bungartz, H.-J.; Lederer, H. The ELPA Library: Scalable Parallel Eigenvalue Solutions for Electronic Structure Theory and Computational Science. *J. Phys.: Condens. Matter* **2014**, *26* (21), 213201. https://doi.org/10.1088/0953-8984/26/21/213201.
(4) Yu, V. W.; Corsetti, F.; García, A.; Huhn, W. P.; Jacquelin, M.; Jia, W.; Lange, B.; Lin, L.; Lu, J.; Mi, W.; Seifitokaldani, A.; Vázquez-Mayagoitia, Á.; Yang, C.; Yang, H.; Blum, V. ELSI: A Unified Software Interface for Kohn–Sham Electronic Structure Solvers. *Computer Physics Communications* **2018**, *222*, 267–285. https://doi.org/10.1016/j.cpc.2017.09.007.
(5) Perdew, J. P.; Burke, K.; Ernzerhof, M. Generalized Gradient Approximation Made Simple. *Phys. Rev. Lett.* **1996**, *77* (18), 3865–3868. https://doi.org/10.1103/PhysRevLett.77.3865.
(6) Perdew, J. P.; Burke, K.; Ernzerhof, M. Generalized Gradient Approximation Made Simple [Phys. Rev. Lett. 77, 3865 (1996)]. *Phys. Rev. Lett.* **1997**, *78* (7), 1396–1396. https://doi.org/10.1103/PhysRevLett.78.1396.
(7) Hermann, J.; Tkatchenko, A. Density Functional Model for van Der Waals Interactions: Unifying Many-Body Atomic Approaches with Nonlocal Functionals. *Phys. Rev. Lett.* **2020**, *124* (14), 146401. https://doi.org/10.1103/PhysRevLett.124.146401.
(8) Lenthe, E. van; Baerends, E. J.; Snijders, J. G. Relativistic Regular Two-component Hamiltonians. *J. Chem. Phys.* **1993**, *99* (6), 4597–4610. https://doi.org/10.1063/1.466059.
(9) Heyd, J.; Scuseria, G. E.; Ernzerhof, M. Hybrid Functionals Based on a Screened Coulomb Potential. *J. Chem. Phys.* **2003**, *118* (18), 8207–8215. https://doi.org/10.1063/1.1564060.
(10) Krukau, A. V.; Vydrov, O. A.; Izmaylov, A. F.; Scuseria, G. E. Influence of the Exchange Screening Parameter on the Performance of Screened Hybrid Functionals. *J. Chem. Phys.* **2006**, *125* (22), 224106. https://doi.org/10.1063/1.2404663.
(11) Stukowski, A. Visualization and Analysis of Atomistic Simulation Data with OVITO–the Open Visualization Tool. *Modelling and Simulation in Materials Science and Engineering* **2010**, *18* (1), 015012. https://doi.org/10.1088/0965-0393/18/1/015012.
(12) Blum, V.; Gehrke, R.; Hanke, F.; Havu, P.; Havu, V.; Ren, X.; Reuter, K.; Scheffler, M. Ab Initio Molecular Simulations with Numeric Atom-Centered Orbitals. *Computer Physics Communications* **2009**, *180* (11), 2175–2196. https://doi.org/10.1016/j.cpc.2009.06.022.
(13) Haldar, R.; Kozlowska, M.; Ganschow, M.; Ghosh, S.; Jakoby, M.; Chen, H.; Ghalami, F.; Xie, W.; Heidrich, S.; Tsutsui, Y.; Freudenberg, J.; Seki, S.; Howard, I. A.; Richards, B. S.; Bunz, U. H. F.; Elstner, M.; Wenzel, W.; Wöll, C. Interplay of Structural Dynamics and Electronic Effects in an Engineered Assembly of Pentacene in a Metal–Organic Framework. *Chem. Sci.* **2021**, *12* (12), 4477–4483. https://doi.org/10.1039/D0SC07073D.
(14) Haldar, R.; Fu, Z.; Joseph, R.; Herrero, D.; Martín-Gomis, L.; Richards, B. S.; Howard, I. A.; Sastre-Santos, A.; Wöll, C. Guest-Responsive Polaritons in a Porous Framework: Chromophoric Sponges in Optical QED Cavities. *Chem. Sci.* **2020**, *11* (30), 7972–7978. https://doi.org/10.1039/D0SC02436H.
(15) Coropceanu, V.; Cornil, J.; da Silva Filho, D. A.; Olivier, Y.; Silbey, R.; Brédas, J.-L. Charge Transport in Organic Semiconductors. *Chem. Rev.* **2007**, *107* (4), 926–952. https://doi.org/10.1021/cr050140x.
(16) Tkatchenko, A.; Scheffler, M. Accurate Molecular Van Der Waals Interactions from Ground-State Electron Density and Free-Atom Reference Data. *Physical Review Letters* **2009**, *102* (7). https://doi.org/10.1103/PhysRevLett.102.073005.

(17) Winkler, C.; Mayer, F.; Zojer, E. Analyzing the Electronic Coupling in Molecular Crystals—The Instructive Case of α-Quinacridone. *Advanced Theory and Simulations* **2019**, *2* (5), 1800204. https://doi.org/10.1002/adts.201800204.
